# Supplementary material for: Genome-scale metabolic models consistently predict in vitro characteristics of Corynebacterium striatum
Source: Front Bioinform. 2023 Oct 23;3:1214074. doi: 10.3389/fbinf.2023.1214074 (PMC10626998; doi:10.3389/fbinf.2023.1214074)
Supplement: Supplementary file 1 [file DataSheet1.PDF]

Supporting Information

Media preparation

LB (Sigma), BHI (Roth), TSB (Oxoid), and RPMI (Thermo Fischer[51]) powders were dissolved in MilliQ water. Afterward, the media were sterilized by autoclaving or by 0.22 µm filtration for RPMI.

M9 Minimal Salts were ordered from Sigma (M6030)[53]. The M9 medium was then prepared using a 5× autoclaved stock of the M9 salts with the addition of 1 mM $MgSO_4 \cdot 7\,H_2O$, 0.1 mM $CaCl_2 \cdot 2\,H_2O$, 0.1 % D-glucose which were all sterilized by filtering through a 0.22 µm filter.

CGXII was prepared according to the protocol given by Unthan et al. 55. All components were prepared as stock solutions in water, and sterilized by filtering through a 0.22 µm filter. The final medium was then prepared by mixing the stock solutions, adding water to fill to the desired volume, and adjusting the pH to 7 using a 1 M NaOH solution. Afterward, the medium was sterilized by filtering.

All tested additives were prepared as stock solutions, sterilized by filtering, and then added to obtain the desired concentration as shown in Table S1. The trace element solution contains $MnCl_2 \cdot 4\,H_2O$, $ZnCl_2$, $CuCl_2 \cdot 2\,H_2O$, $CoCl_2 \cdot 6\,H_2O$, $Na_2MoO_4 \cdot 2\,H_2O$, H_3BO_3 and $NiCl_2 \cdot 6\,H_2O$.

Table S1 | Metabolites that were used as additives for growth enhancement. This table holds the concentration that was used if the metabolite was added and the paper that this concentration was taken from.

**metabolite**	**concentration** [g/L]	**based on**
Nicotinate	0.001	Yao et al. (2018)
(R)-Pantothenate	0.001	Yao et al. (2018)
Co^{2+}	0.0024	Taylor and Holland (1989)
L-Cysteine	0.05	Concentration in RPMI[51]
Fe^{2+}	0.01	Concentration in CGXII[55]

Growth in liquid medium

All doubling times were extracted by fitting a logistic equation to the growth data. Growth curves with extracted doubling times in TSB are shown in Figure S1.

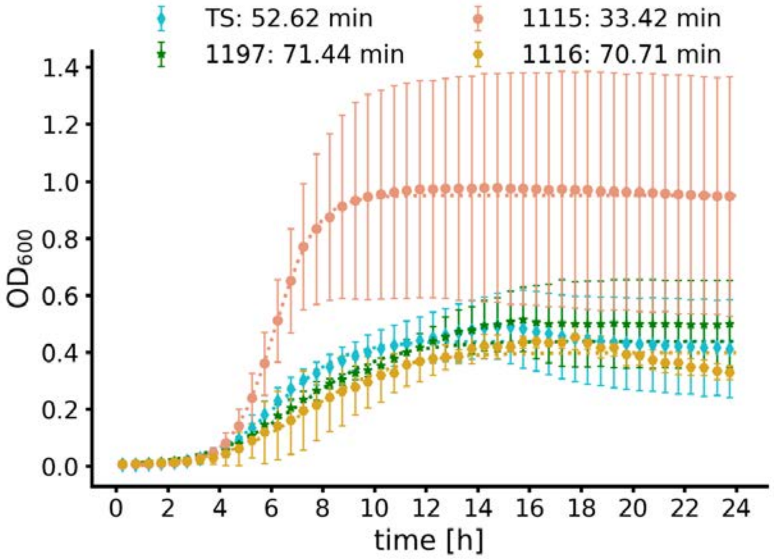

Figure S1 | *In vitro* growth curve in TSB of the strains TS, 1197, 1115 and 1116. Growth *in vitro* in TSB was determined via OD600 measurements for 24 h (data points). Shown are the means of $n = 3$ independent experiments with standard deviation. Logistic fit was then calculated using `curve_fit` (dotted lines). The doubling times extracted from the logistic fit are indicated in the legend. For strain TS and 1197 we only fitted data up to 12.5h to avoid fitting to diphasic growth.

Model status

Table S2 | Overview on key numbers of all models. Strains TS, 1197, 1115 and 1116 were compared to laboratory work. Strain KC was not available for laboratory experiments.

strain	TS	1197	1115	1116	KC
# metabolites	1,382	1,141	1,197	1,212	1,053
# reactions	2,002	1,674	1,750	1,788	1,541
# genes	772	756	763	755	719
metabolic coverage [in %]	2.59	2.21	2.29	2.37	2.14
MEMOTE score [in %]	86.4	86.7	86.2	86.3	84.1

In silico media composition

Table S3 | *In silico* composition of the different media used in this study. Indicated are the BiGG IDs of the metabolites for which exchanges were opened during simulation.

LB		RPMI		M9	CGXII
glc__D	hxan	ca2	pnto__R	ca2	nh4
ala__L	dcyt	no3	fol	cl	so4
asp__L	thymd	mg2	pydxn	glc__D	k
glu__L	ura	so4	cbl1	h2o	pi
his__L	uri	cl	glc__D	h	urea
leu__L	dad_2	k	gthrd	k	ca2
met__L	adn	na1	h2o	mg2	cl
pro__L	chor	pi	o2	na1	mg2
thr__L	o2	gly	hco3	nh4	fe2
tyr__L	h	arg__L	4hpro_LT	o2	mn2
arg__L	h2o	asn__L	b12	pi	ni2
cys__L	amp	asp__L	co2	so4	cu2
gly	cmp	cys__L	h		zn2
ile__L	cro4	glu__L			btn
lys__L	cobalt2	gln__L			glc__D
phe__L	cu2	his__L			h2o
ser__L	fe2	ile__L			o2
trp__L	fe3	leu__L			na1
val__L	gmp	met__L			h
thm	gsn	phe__L			34dhbz
ribflv	h2s	pro__L			
pnto__R	lipoate	ser__L			
pydx	mn2	thr__L			
btn	mobd	trp__L			
fol	nac	tyr__L			
cbl1	nh4	val__L			
cbl2	ni2	lys__L			
b12	pheme	btn			
na1	ump	chol			
cl	zn2	ncam			
so4	aso3	4abz			
k	cd2	ribflv			
pi	hg2	thm			
ca2	ins	inost			
mg2					

Escher map of strain 1116

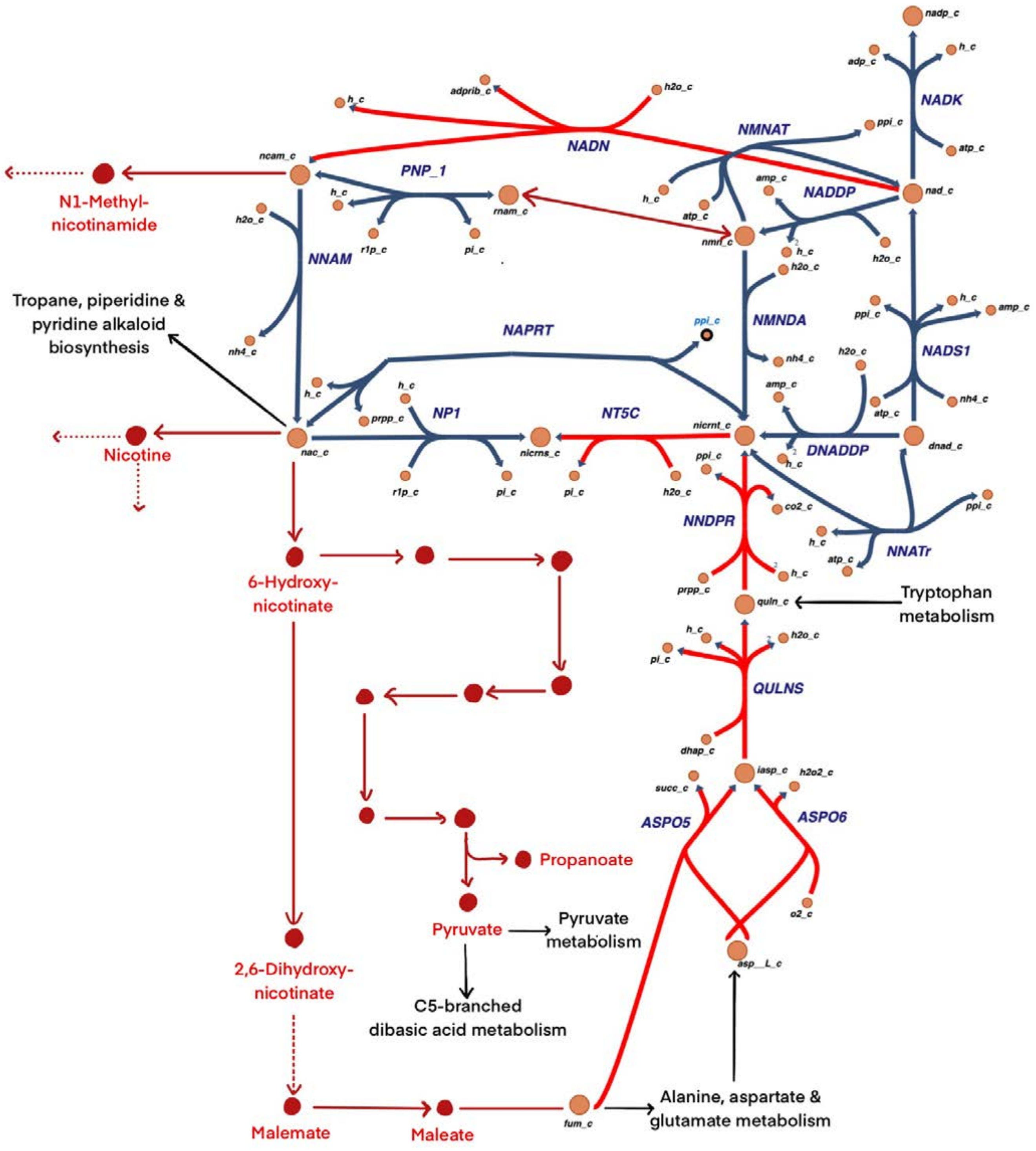

Figure S2 | Escher[24] map of the nicotinate metabolism. Links to other pathways are shown on the map in black. Missing reactions from strain 1116 are shown in red.
